# Positive feedback regulation between IL10 and EGFR promotes lung cancer formation

**DOI:** 10.18632/oncotarget.7894

**Published:** 2016-03-03

**Authors:** Tsung-I Hsu, Yi-Chang Wang, Chia-Yang Hung, Chun-Hui Yu, Wu-Chou Su, Wen-Chang Chang, Jan-Jong Hung

**Affiliations:** ^1^ Center for Infection Disease and Signal Research, College of Medicine, Tainan, Taiwan; ^2^ Institute of Bioinformatics and Biosignal Transduction, College of Bioscience and Biotechnology, National Cheng Kung University, Tainan, Taiwan; ^3^ The Ph.D. Program for Neural Regenerative Medicine, College of Medical Science and Technology, Taipei Medical University and National Health Research Institutes, Taipei, Taiwan; ^4^ Institute of Basic Medical Sciences, College of Medicine, National Cheng Kung University, Tainan, Taiwan; ^5^ Department of Pharmacology, College of Medicine, National Cheng Kung University, Tainan, Taiwan; ^6^ Department of Internal Medicine, College of Medicine and Hospital, National Cheng Kung University, Tainan, Taiwan; ^7^ Graduate Institute of Medical Sciences, College of Medicine, Taipei Medical University, Taipei, Taiwan

**Keywords:** IL10, Src, nucleolin, EGFR

## Abstract

The role of IL10 in the tumorigenesis of various cancer types is still controversial. Here, we found that increased IL10 levels are correlated with a poor prognosis in lung cancer patients. Moreover, IL10 levels were significantly increased in the lungs and serum of EGFR^L858R^- and Kras4b^G12D^-induced lung cancer mice, indicating that IL10 might facilitate lung cancer tumorigenesis. IL10 knockout in EGFR^L858R^ and Kras4b^G12D^ mice inhibited the development of lung tumors and decreased the levels of infiltrating M_2_ macrophages and tumor-promoting T_reg_ lymphocytes. We also showed that EGF increases IL10 expression by enhancing IL10 mRNA stability, and IL10 subsequently activates JAK1/STAT3, Src, PI3K/Akt, and Erk signaling pathways. Interestingly, the IL10-induced recruitment of phosphorylated Src was critical for inducing EGFR through the activation of the JAK1/STAT3 pathway, suggesting that Src and JAK1 positively regulate each other to enhance STAT3 activity. Doxycycline-induced EGFR^L858R^ mice treated with gefitinib and anti-IL10 antibodies exhibited poor tumor formation. In conclusion, IL10 and EGFR regulate each other through positive feedback, which leads to lung cancer formation.

## INTRODUCTION

Lung cancer is a disease that presents high incidence and mortality rates, and it is primarily associated with genetic mutations that affect the EGFR, KRAS, and EML4-ALK fusion proteins [1]. Recently, the formation of a microenvironment enriched in cancer-associated fibroblasts, macrophages, and regulatory T (T_reg_) cells has been proposed as a key step in tumor development mediated by secretions of chemokines and cytokines [2]. However, evidence supporting the requirement of epithelium-derived cytokines for the development of epithelial tumors is lacking, although IL8 secretion by lung cancer cells has been demonstrated [3]. Interleukin 10 (IL10), a T_H_2 cytokine secreted by almost all leukocytes, regulates immune activity by suppressing inflammatory responses, including antigen presentation, monocyte and macrophage functions, and phagocytosis [4–6]. Moreover, IL10 favors the differentiation of CD4^+^ cells toward T_reg_ [7] and monocytes toward M_2_-like macrophages [8], which accumulate in the tumor microenvironment [9]. Furthermore, IL10 levels obviously increase in cancer patients [10–14], and IL10 expression by tumor-associated macrophages is correlated with a poor prognosis [15]. Upregulation of IL10 in cancer is probably caused by genetic polymorphism in the IL10 promoter [16], suggesting that an increase of IL10 is essential for tumor development. However, the mechanism of IL10 upregulation is not well understood, and it remains unclear whether cancer cells secrete IL10 and whether IL10 has an impact on the aggressiveness and malignancy of cancer cells.

IL10 binds to IL10 receptor subunit alpha (IL10RA) to trigger the Janus kinase 1 (JAK1)/signal transducer and activator of transcription 3 (STAT3)-mediated signaling pathway in macrophages and non-macrophages [6]. IL10-bound IL10RA induces JAK1 to phosphorylate IL10RA at Y446 and Y496, and STAT3 associates with phospho-IL10RA for the subsequent phosphorylation by JAK1. Phospho-STAT3 translocates to the nucleus and executes transcription [17]. Because the inhibition of JAK1/STAT3 signaling exhibits potent tumor-suppressive effects on lung cancer [18], it is important to study how IL10 induces JAK1/STAT3 signaling in cancer development. Here, we establish two spontaneously Kras4b^G12D^ and EGFR^L858R^-induced lung cancer mice to illustrate the development of lung cancer and the tumor microenvironment. Knocking out IL10 in these two mice prevented lung cancer formation and provided direct evidence for the role of IL10 in lung cancer formation.

## RESULTS

### Accumulation of IL10 is associated with poor prognosis in human lung cancer

**Figure 1:**
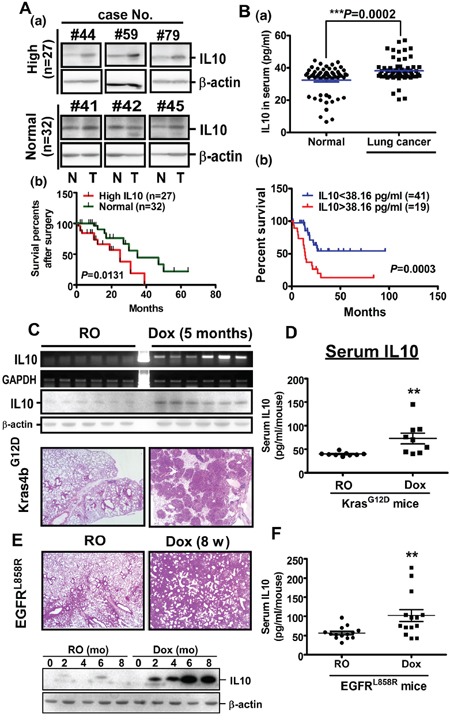
IL10 is over expressed in lung cancer **A.** Upper panel: the representative image of IL10 expression from 6 independent groups. The human specimens were homogenized for Western blotting targeting IL10. Lower panel: The relevance between survival rate and IL10 level from 27 and 32 lung cancer patients with higher and unaltered IL10, respectively. **B.** Upper panel: IL10 level in human serum was measured by ELISA. Data were expressed as mean±s.e.m. Lower panel: The average serum concentration of IL10, 38.16 pg/ml, was set as the cutting edge to evaluate the correlation with prognosis. **C, E.** IL10 level in Kras4b^G12D^- and EGFR^L858R^-induced lung cancer mice. Lungs from mice were prepared for RT-PCR, Western blotting and HE staining. **D, F.** IL10 levels in the serum from Kras4b^G12D^ and EGFR^L858R^ mice with or without lung cancer were measured by ELISA.

To understand the role of IL10 in lung cancer, we studied IL10 levels in normal and lung tumor tissues (Figure 1A and Supplementary Figure S1). Among 59 lung cancer patients, 27 patients exhibited higher IL10 levels, whereas the remaining 32 patients showed unaltered IL10 levels in their lung tumor tissues. Patients with higher IL10 expression presented lower survival rates (Figure 1A). In the lung tumor tissues, IL10 accumulated in the epithelial cells and CD68^+^ macrophages, suggesting that IL10 from cancer cells and tumor-associated macrophages is involved in lung cancer progression (Supplementary Figure S1). In addition, because IL10 is a secreted protein, we studied the IL10 levels in the sera of 60 normal individuals and 60 lung cancer patients (Figure 1B). The data indicated that the IL10 levels in the sera of the lung cancer patients (38.16 pg/mL) were significantly higher than that in the normal individuals (32.55 pg/mL) (Figure 1B, upper panel). Furthermore, among the 60 lung cancer patients, the average concentration of IL10 was obviously higher in stages III & IV than in stages I & II (Supplementary Table S1), implying an involvement of IL10 in tumor progression. Importantly, 19 patients with IL10 levels higher than 38.16 pg/mL exhibited a poorer prognosis relative to the other patients (Figure 1B, lower panel). To examine the molecular mechanism of IL10 in lung cancer formation, we studied IL10 expression in Kras4b^G12D^- and EGFR^L858R^-induced lung cancer mice. After doxycycline treatment, pulmonary tumor nodules were observed on the lung surfaces, and IL10 mRNA and protein levels were increased in parallel (Figure 1C and 1E). In addition, IL10 was increased in the serum of doxycycline-induced Kras4b^G12D^- and EGFR^L858R^ mice compared with that of normal individuals (Figure 1D and 1F). In summary, these results from clinical cohorts and animal models show that IL10 levels were highly upregulated in lung cancer tissues and correlated with a poorer patient prognosis.

### IL10 secretion increased by the EGF/PI3K/nucleolin axis promotes the proliferation of lung tumor cells

**Figure 2:**
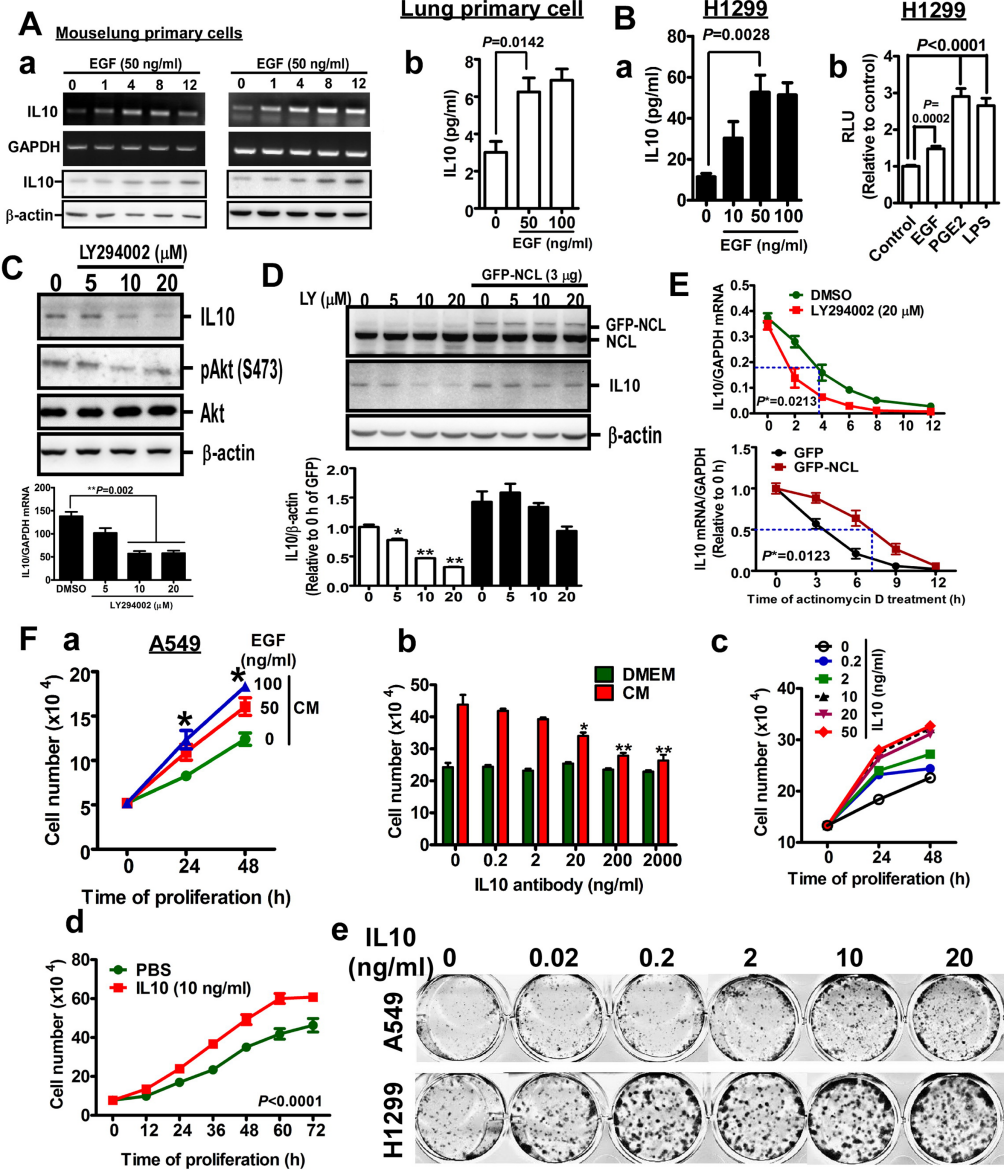
EGF induces IL10 expression, and IL10 increases proliferation **A (a).** After treatment with EGF for 24h in serum-free media, mouse lung primary cells were harvested for RT-PCR and Western blotting targeting IL10. **(b).** The medium was collected for detecting IL10 by ELISA. **B (a).** IL10 secretion in EGF-treated H1299 cells **(b).** H1299 cells-expressed pGL2-IL10 promoter were harvested for luciferase reporter assay after EGF treatment. PGE_2_ and LPS treatments are positive controls. Data were expressed as mean±s.e.m. **C.** After LY294002 treatment for 24h, H1299 cells were harvested for Western blotting to detect IL10. The result was quantified as lower panel. **D.** GFP-nucleolin (NCL)-overexpressed cells were treated with LY294002 for 24h, and harvested for Western blotting. Lower panel is the quantified result. Data were expressed as mean±s.e.m. (*p<0.05, **p<0.01). **E.** After treatment with LY294002 or GFP-NCL overexpression for 24h, cells were treated with actinomycin D for indicated time interval. Total RNA was extracted for RT-qPCR targeting IL10. **F (a).** A549 cells were treated with medium from cells treated with EGF for 24h, cell number was counted by hemacytometer. Data were expressed as mean±s.e.m. (*p<0.05). **(b).** Cells were treated with conditional media in the absence or presence of anti-IL10 antibody for 24h. Cell number was counted. **(c).** After treatment with recombinant IL10 for 24h, cell number was counted. **(d).** After 10 ng/ml IL10 treatment for indicated time, cell number was counted. **(e).** Cells were cultured in the presence of indicated dose of recombinant IL10 protein for 14 days. Colony was stained by methyl-blue.

In Figure 1, we show that IL10 increases in lung tumors and the serum of Kras4b^G12D^ and EGFR^L858R^-induced lung cancer mice. Therefore, we performed *in vitro* experiments in which cells were treated with EGF to turn on EGFR-mediated signaling to mitigate lung tumorigenesis *in vivo*. EGF treatment *in vitro* represents Kras4b^G12D^ and EGFR^L858R^-induced signaling *in vivo* in lung cancer mice. In mouse primary lung cells, EGF increased the expression and secretion of IL10 (Figure 2A), and in the EGF-treated lung cancer cells, IL10 secretion was also increased. Consistent with previous studies in which PGE_2_ and LPS were reveal to induce transcriptional activity of IL10 [19], here we found that EGF increased transcriptional activity of IL10, indicating that EGF induces IL10 expression by enhancing transcription (Figure 2B). Various inhibitors, such as FTI-276, U0126, LY294002, and MK2206, were then used to study the molecular mechanism underlying IL10 induction by EGF. The data indicated that the inhibition of PI3K activity by LY294002 significantly reduced IL10 expression (Figure 2C), and a microarray analysis revealed that nucleolin overexpression increased the expression of several interleukins, including IL10 (Supplementary Figure S2A), which is likely because PI3K has been reported to regulate the RNA-protecting ability of nucleolin [20]. Therefore, nucleolin knockdown inhibited IL10 expression, whereas GFP-nucleolin overexpression increased IL10 levels (Supplementary Figure S2A) and rescued IL10 levels inhibited by LY294002 (Figure 2D). Previous studies revealed that nucleolin could be recruited by and increase the stability of RNA [20]. Here, we found that LY294002 decreased IL10 RNA stability and GFP-nucleolin overexpression increased mRNA stability, indicating that nucleolin increases IL10 expression by stabilizing its mRNA (Figure 2E). To study the role of secreted IL10 in the proliferation of cancer cells, we collected the conditioned media of A549 cells treated with EGF in serum-free media for 24 h. After serum starvation for 12 h, the cells were treated with conditioned media or serum-free media. As shown in Figure 2F(a), the conditioned media from EGF-treated cells significantly increased cancer cell proliferation in a dose-dependent manner, and this phenomenon was attenuated by the presence of IL10 antibodies in the conditioned media (Figure 2F(b)), suggesting that IL10 in the media is required for EGF-induced proliferation. This result prompted us to study whether recombinant IL10 affects cancer cells proliferation. As shown in Figure 2F(c), IL10 increased proliferation in a dose- and time-dependent manner (Figure 2F(c, d)), and lung cancer cell colony formation was obviously enhanced by IL10 (Figure 2F(e)). Thus, these results show that EGF-induced IL10 expression and secretion is important for EGF-induced proliferation.

### *IL10* knockout in Kras4b^G12D^- and EGFR^L858R^-induced lung cancer mice inhibits cancer formation

**Figure 3:**
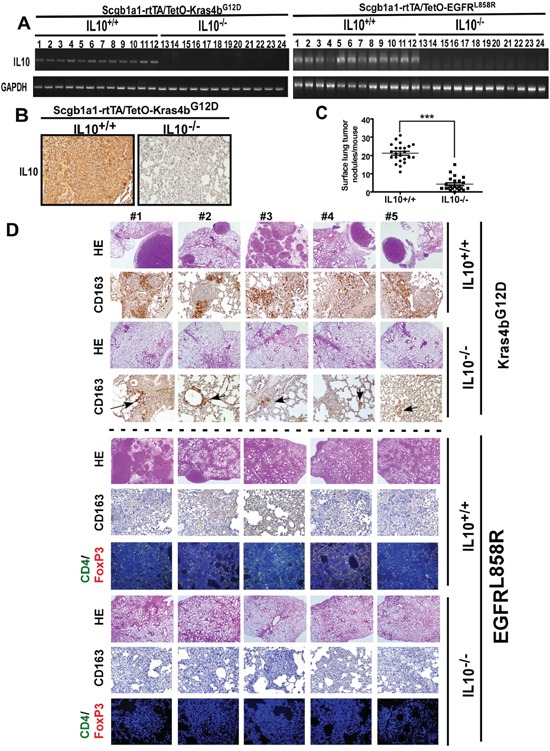
IL10-deficent mice failed to develop lung cancer induced by Kras4bG12D and EGFRL858R **A.** The mRNA level of IL10 in indicated transgenic mice. **B.** The protein level of IL10 was detected by IHC. **C.** After doxycycline administration for 5 months, lungs of Kras4b^G12D^ mice were excised for counting surface tumor nodules. **D.** Lungs excised from doxycycline-treated Kras4b^G12D^ (5 months) and EGFR^L858R^ (1 month) were immunohistochemically stained by HE and the anti-CD163 antibody, and immunofluorescently stained by anti-CD4 and anti-FoxP3 antibodies. Brown and grey in slides of Kras4b^G12D^ and EGFR^L858R^, respectively, represent CD163^+^ cells.

Although the *in vivo* role of IL10 in lung cancer has not been previously elucidated, in this study, we showed that IL10 was highly increased in lung cancer, and EGF promoted proliferation by increasing IL10 secretion. Therefore, we attempted to investigate whether IL10 affects lung cancer development *in vivo*. IL10-deficient Kras4b^G12D^- and EGFR^L858R^-induced lung cancer mice (*Scgb1a1-rtTA/TetO-Kras4b^G12D^/IL10*^−/−^ and *Scgb1a1-rtTA/TetO-EGFR^L858R^/IL10^−/−^*) were established for this purpose (Figure 3). Initially, we confirmed that IL10 mRNA and protein were not present in these IL10-deficient mice (Figure 3A, 3B). Analysis of the histological staining and nodule numbers showed that these *IL10* knockout mice failed to develop significant lung tumors (Figure 3C, 3D); thus, these data on Kras4b^G12D^- and EGFR^L858R^-induced lung cancer provide direct evidence that IL10 knockout inhibits lung cancer formation. Because IL10 is known to regulate the differentiation of M_2_ macrophages and T_reg_ cells in the microenvironment [7, 9], we first evaluated whether *IL10* knockout inhibits the formation of the microenvironment. As shown in Figure 3D, lung tumors in the Kras4b^G12D^ and EGFR^L858R^ mice exhibited thriving clusters of CD163^+^ cells representing M2 macrophages, and an accumulation of CD4^+^/FoxP3^+^ cells was only observed in the lung tumors of EGFR^L858R^ mice, thus representing T_reg_ cells. However, IL10 knockout inhibited the accumulation of M_2_ macrophages and T_reg_ cells (Figure 3D). Based on these results, the EGFR-pathway appears to be more important for the formation of the tumor microenvironment than the Kras pathway, and IL10 appears to be crucial for the EGFR^L858R^-mediated tumor microenvironment.

**Figure 4:**
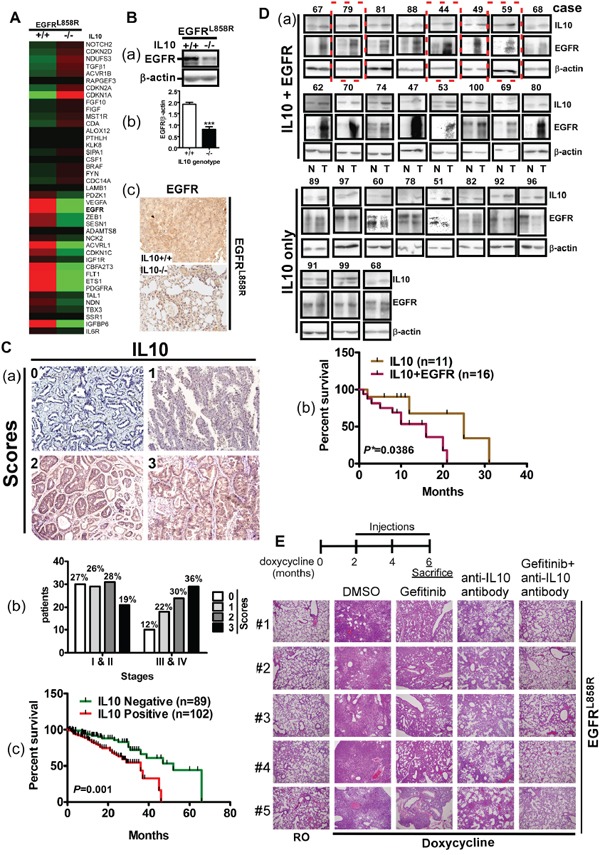
IL10 regulates EGFR expression through Src activation **A.** The proliferation-related gene expression profile from microarray analysis in EGFR^L858R^ and EGFR^L858R^/IL10^−/−^. **B (a).** Lung extracts were homogenized for detecting EGFR expression by Western blotting, and the signal of EGFR was quantified. **(b).** Data was expressed as mean±s.e.m. (***p<0.001). **(c).** The histological slides of lungs were stained for EGFR by IHC. **C (a).** Four scores to define IL10 expression detected by IHC in 192 lung cancer patients. **(b).** The IL10 expression in stages I&II and III&IV. **(c).** Negative (scores 0 and 1) and positive (scores 2 and 3) IL10 expression was correlated with survival period of patients. **D.** EGFR expression was detected in normal and tumor parts in 27 IL10-positive patients. **(a)**. Upper panel is the images of Western blotting for EGFR. **(b).** Lower panel is the correlation of survival with IL10-positive/EGFR overexpression (IL10+EGFR) and IL10-positive only (IL10). **E.** Upper panel is the schedule of injection with the indicated drug. Lower panel: EGFR^L858R^ mice were injected with gefitinib with or without anti-IL10 antibody injection for 4 weeks. Lungs were excised for HE staining.

Next, we studied the effects of IL10 on lung cancer itself (Figure 4). The gene expression profiles of lungs in the EGFR^L858R^ and EGFR^L858R^/IL10^−/−^ mice, which were administered doxycycline for 1 month, were analyzed by a microarray analysis. After performing a gene set enrichment analysis (GSEA), we clustered the proliferation-related genes and found that EGFR was dramatically decreased by *IL10* knockout (Figure 4A). We subsequently confirmed that *IL10* knockout reduced EGFR protein levels, implying that IL10 is involved in the regulation of EGFR expression (Figure 4B). Analyzing the relationship between IL10 levels and lung cancer progression, we found that late-stage patients showed higher IL10 expression and patients with higher IL10 levels presented lower survival rates (Figure 4C and Table 1). Furthermore, an analysis of 27 lung cancer patients exhibiting high IL10 expression levels showed that 16 of these patients also exhibited EGFR overexpression (Figure 4D(a)). A comparison of the survival rates among patients with IL10 overexpression only and patients with IL10 and EGFR overexpression revealed that the patients with IL10 and EGFR overexpression presented poorer prognoses (Figure 4D(b)). Therefore, we speculate that the simultaneous inhibition of IL10 and EGFR may achieve greater anti-tumor effects compared with the individual inhibition of IL10 or EGFR. To evaluate the effect of combining an EGFR inhibitor with an anti-IL10 antibody on lung cancer formation, EGFR^L858R^ mice that had developed lung tumors were administered gefitinib and the anti-IL10 antibody by intraperitoneal injection (Figure 4E). Compared with mice supplied with RO water, doxycycline dramatically induced lung tumor formation, which diffusely invade the pulmonary parenchyma including the alveoli and trachea. In particular, gefitinib and the anti-IL10 antibodies were individually effective at reducing the amount of tumor cells, and the combination of gefitinib with the anti-IL10 antibody further inhibited lung cancer formation (Figure 4E), suggesting that neutralizing IL10 synergistically blocks lung cancer malignancy through EGFR inhibition. However, an investigation to determine whether IL10 expression correlates with EGFR mutations in the 191 patients used to analyze IL10 expression by IHC showed that only 39 of these patients were examined for EGFR status. In particular, among the 19 IL10-positive patients, 8 exhibited WT EGFR and 11 exhibited EGFR mutations, whereas among the 20 IL10-negative patients, 8 exhibited WT EGFR and 12 exhibited EGFR mutations (Supplementary Table S2). A statistical analysis indicated that IL10 expression was not correlated with the EGFR mutations, including exon 19 deletion and L858R (Supplementary Table S2). Because KRAS mutations are not common in Taiwan, the KRAS status was not examined in any patients in the present study. Moreover, both IL10 and EGFR enhance lung cancer formation, and IL10 might increase EGFR expression. In conclusion, IL10 appears to have a positive effect on the formation of the tumor microenvironment via M_2_ macrophages as well as through the expression of EGFR in cancer cells, thereby enhancing lung cancer formation.

**Table 1:** Characteristics of lung cancer patients, which are involved in determining IL10 expression by IHC and Western blotting

Characteristics	IL10 negative	IL10 positive	*p*-value
**Patients (n)**	89 (46.6%)	102 (53.4%)	
**Gender**			
Male	49 (47.6%)	54 (52.4%)	0.0655^a^
Female	50 (56.8%)	38 (43.2%)	
**Age (mean±s.e.m)**	65.15±0.8	65.1±0.7	0.9623^b^
**ADC^1^**	74 (52.9%)	66 (47.1%)	0.1696^a^
**SCC^2^**	15 (39.5%)	23 (60.5%)	
**LCC^3^**	1 (25%)	3 (75%)	
**Stages**			
I&II	59 (53.2%)	52 (46.8%)	0.0389*^a^
III&IV	28 (34.6%)	53 (65.4%)	
**Recurrence**	9 (33.3%)	18 (66.7%)	
**EGFR mutations^#^**	12 (52.2%)	11 (47.8%)	
**Phospho-Src (Y419)**	Not determined	Negative: 44 (43.1%)	
		Positive: 58 (56.9%)	
**EGFR expression**	Not determined	Normal: 11 (40.7%)	
		Overexpression: 16 (59.3%)	

Abbreviation:

1 adenocarcinoma;

2 squamous cell carcinoma;

3 large cell carcinoma

# EGFR mutations include exon 19 deletion and L858R of exon 21.

a Fisher's extract test,

b Student's t test

### IL10-mediated Src recruitment to IL10RA facilitates Src and JAK1 co-regulation to induce EGFR expression

**Figure 5:**
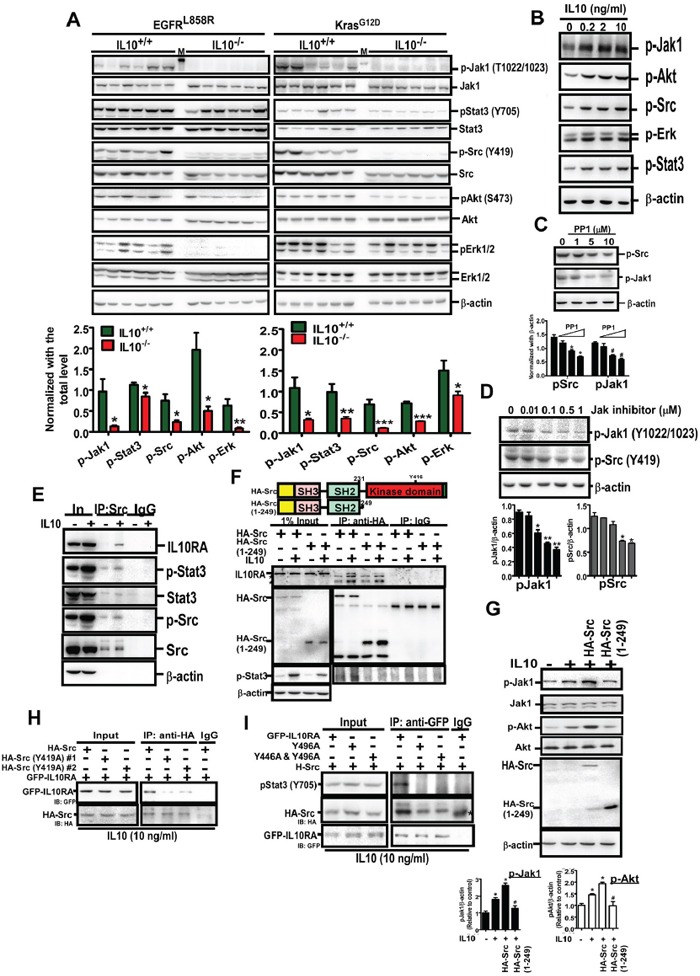
IL10 induces oncogenic signaling through inducing the interaction of IL10RA with phospho-Src **A.** Effect of IL10 knockout on EGFR^L858R^ and Kras4b^G12D^-induced signaling. Lungs were homogenized for Western Blotting using the indicated antibody. Lower panel is the quantitative result. Data were expressed as mean±s.e.m. (*p<0.05, **p<0.01, ***p<0.001). **B, C, D.** After treatment with IL10 for 24 h, cell harvests were prepared for Western blotting with indicated antibodies. The lower panel of (C) and (D) is quantitative result. **E.** The immune-complex immunoprecipitated by the anti-Src antibody was analyzed by Western blotting using the indicated antibody. **F.** After transfection with wild-type HA-Src or kinase-dead HA-Src (aa 1-249), for 24h, H1299 cells with or without IL10 treatment were harvested for immunoprecipitation by the anti-HA antibody, and analyzed by Western blotting. Upper panel is the domain illustration of HA-Src. **G.** After transfection of pHA-Src and pHA-Src(1-249), and IL10 treatment, cellular extracts were prepared for Western blotting using indicated antibodies. Lower panel is the quantitative result for phospho-JAK1 and phospho-Akt. Data were expressed as mean±s.e.m. *p<0.05. **H, I.** After transfection with indicated plasmids and IL10 treatment, cellular extracts were prepared for immunoprecipitation using the anti-HA or anti-GFP antibody. The immunoprecipitated complex was analyzed by Western blotting.

Based on the involvement of IL10 in the formation of tumor microenvironment and tumor development, we studied how IL10 increases EGFR expression (Figure 5). The IL10 knockout significantly decreased the levels of p-JAK1 (T1022/1023), p-STAT3 (Y705), p-Src (Y419), p-Akt (S473), and p-Erk, implying that IL10 promotes lung cancer formation by activating these pathways (Figure 5A). In the H1299 cells, IL10 obviously increased the phosphorylation of the above mentioned kinases (Figure 5B), suggesting that IL10 is involved in activating oncogenic signaling. JAK1 is a well-known kinase that links a subunit of the activated IL10 receptor (IL10RA) with STAT3 phosphorylation [6]. Moreover, phospho-Src is an important upstream activator of Erk and Akt; thus, it increases cell survival-related signaling [21]. However, Src has not been shown to crosstalk with JAK1 to increase cytokine signaling. As shown in Figure 5C and 5D, the Src inhibitor PP1 significantly inhibited JAK1 phosphorylation, whereas the JAK inhibitor significantly inhibited Src phosphorylation, implying that JAK1 and Src induce co-phosphorylation. Interestingly, we found that the IL10 treatment induced an interaction between phospho-Src and IL10RA (Figure 5E and Supplementary Figure S3A), suggesting that Src is involved in IL10RA/JAK1/STAT3 signaling. Furthermore, after deleting the kinase domain, HA-Src (aa 1-249) did not interact with IL10RA (Figure 5F). Importantly, the wild-type Src (and not the kinase-deleted Src) enhanced IL10-induced JAK1 and Akt phosphorylation (Figure 5G), suggesting that the kinase activity of Src is required to regulate IL10RA signaling. In particular, we identified that the phosphorylation of Src at Y419 was critical for the interaction between Src and IL10RA (Figure 5H), suggesting that Src is phosphorylated before the interaction. However, IL10RA did not affect Src phosphorylation at Y419 with or without IL10 (Supplementary Figure S3B). Moreover, Y446 and Y496 of IL10RA were phosphorylated to activate signaling after the binding of the IL10 ligand [6]. Therefore, we found that the mutation to alanine at Y496 not only abolished the interaction with p-STAT3 but also decreased the association with p-Src (Figure 5I), indicating that IL10-activated IL10RA recruits p-Src through Y496 phosphorylation. These findings indicate that the IL10-induced recruitment of phospho-Src by IL10RA is essential to activate the JAK1/STAT3 signaling pathway. In summary, EGF induces IL10 secretion, and IL10-activated IL10RA recruits p-Src to activate JAK1/STAT3, thus leading to the upregulation of EGFR expression.

### Phospho-Src (Y419)-positivity worsens the prognoses of IL10-positive patients

**Figure 6:**
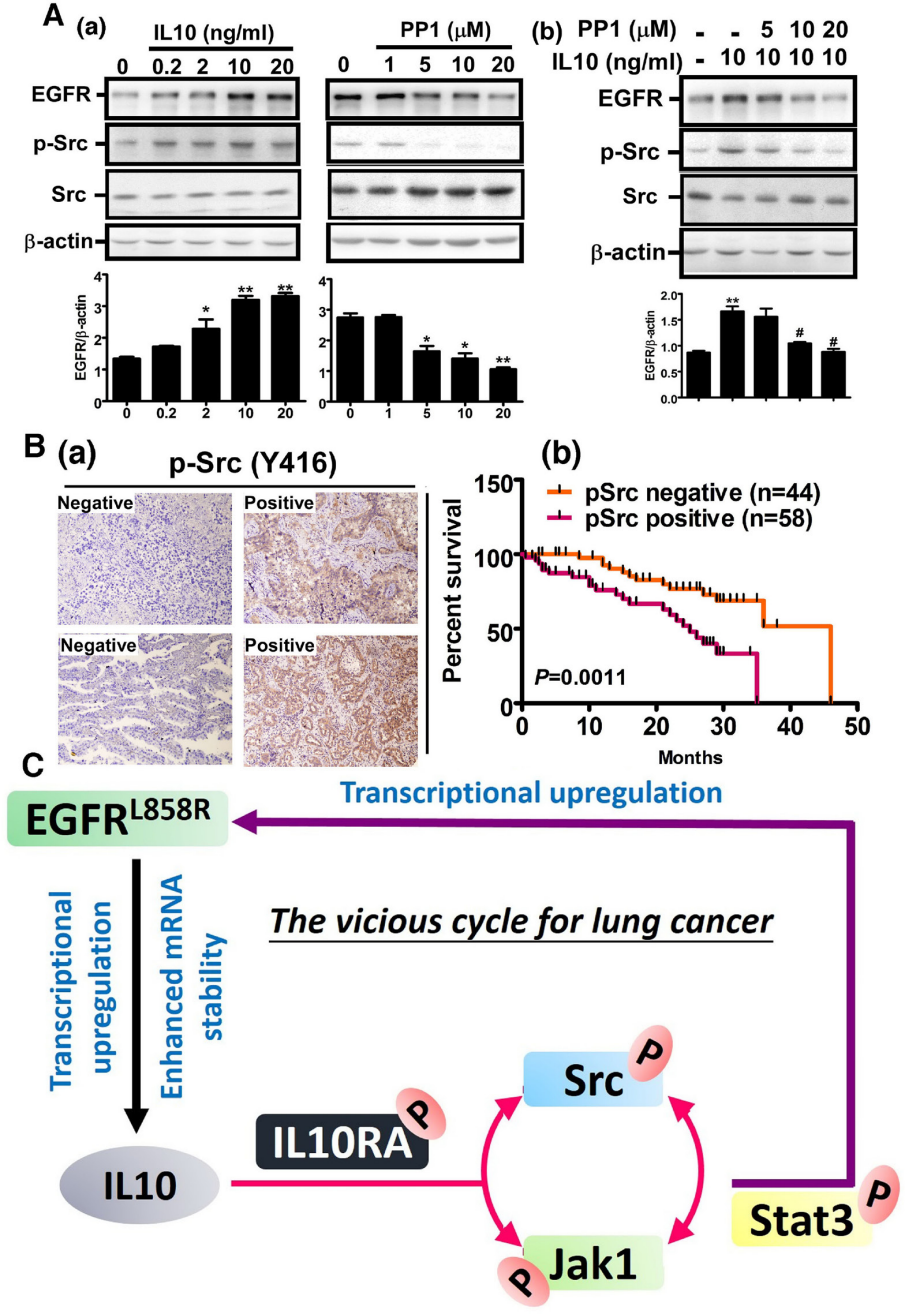
Phospho-Src positivity shortens the survival period of IL10-positive patients **A (a, b).** After IL10 treatment with or without PP1, cell extracts were harvested for Western blotting using antibodies against EGFR, p-Src and β-actin, and the signal was quantified. Data were expressed as mean±s.e.m. *p<0.05 and **p<0.01. **B.** Phospho-Src was detected in 102 IL10-positive patients. Panel **(a)** is representative image of negative and positive phospho-Src by IHC, and panel **(b)** is the correlation of phosphor-Src with prognosis in IL10-positive patients. **C.** The scheme illustrates the positive feedback cycle between EGFR and IL10.

We found that IL10 increased the levels of EGFR and Src phosphorylation in a dose-dependent manner. Conversely, the PP1 treatment with inhibited Src activity decreased EGFR expression (Figure 6A) and reversed the EGFR level induced by IL10, indicating that Src phosphorylation is crucial for IL10-induced EGFR expression (Figure 6A(b)). Finally, we analyzed the relationship between phospho-Src (Y419) and the survival rate of 102 lung cancer patients with positive IL10 expression (Figure 6B). Our data indicate that patients with IL10/phospho-Src-positive expression presented a poorer prognosis. In summary, IL10-induced Src recruitment increases JAK/STAT3 activation, which increases EGFR expression. These results demonstrate that EGFR activation induces IL10 expression by enhancing transcriptional activity and mRNA stability, thereby activating the JAK1-Src cycle to produce a positive feedback for EGFR upregulation (Figure 6C).

## DISCUSSION

In this study, we not only confirmed the upregulation of IL10 and demonstrate its correlation with poor lung cancer prognoses but also showed that knocking out IL10 in two spontaneously induced lung cancer mice, Kras4b^G12D^ and EGFR^L858R^, dramatically prevented lung cancer development *in vivo*. Furthermore, IL10-induced JAK1/STAT3 activity requires phospho-Src recruitment to phospho-IL10RA, which upregulates EGFR expression. Conversely, EGF was found to induce IL10 expression through the PI3K/nucleolin axis. IL10 and EGFR co-regulation produces a vicious cycle for lung cancer development. In the tumor microenvironment, the source of IL10 is variable and may include M_2_ macrophages, lymphocytes, and cancer cells [4, 9]. Previous studies have demonstrated that IL10 acts as a suppressor of the immune system, and an activator of the JAK/STAT3 pathway in cancer cells. Because communication between the tumor microenvironment and tumor cells is critical for cancer development, an experimental model capable of mimicking the tumor environment had to be established to elucidate the role of IL10 in cancer progression. However, the influence of IL10 on tumor development is still controversial. We found that IL10 was increased in Kras4b^G12D^- and EGFR^L858R^-induced lung cancer mice, which is consistent with the results in lung cancer patients and indicates that Kras and EGFR activation are involved in IL10 expression and might be positively related to lung cancer formation. Based on these findings, we established IL10 knockout mice (Kras4b^G12D^/IL10^−/−^ and EGFR^L858R^/IL10^−/−^) to investigate whether IL10 promotes lung cancer formation, and the following results were observed: IL10 is important for the formation of the microenvironment during lung cancer progression, and IL10 and EGFR produce positive feedback regulation that enhances lung cancer tumorigenesis.

First, we found that the IL10 knockout decreases M_2_ macrophages and T_reg_ cells (CD163^+^ and CD4^+^/FoxP3^+^ cells, respectively) suggesting that IL10 is positively involved in the formation of the microenvironment. Previous studies revealed that IL10 promotes cancer development by inhibiting anti-tumor immune responses. In addition to impairing the activation of cytotoxic T lymphocytes (CTLs) and Th_1_ CD4^+^ cells [22], IL10 inhibits the cytolytic activity of natural killer cells and CTLs [23], which are responsible for the immune surveillance of cancer. In addition, the anti-tumor activity of IL10 has been addressed. IL10-deficient mice are sensitive to chemically induced carcinogenesis [24] and spontaneously develop inflammatory bowel disease, which is associated with the occurrence of colon carcinomas [25]. Moreover, IL10 suppresses melanoma tumor growth and metastasis [26]. Our data indicate that IL10 is increased in the tumor microenvironment and *IL10* knockout inhibits the formation of the tumor microenvironment, indicating that IL10 is crucial for the tumor microenvironment. Therefore, IL10 overexpression in M_2_ macrophages may protect cancer cells from lysis by immune cells. Because EGFR activation may trigger the initial step of IL10 synthesis, its secretion out of cancer cells to develop the tumor microenvironment would benefit cancer development. Because the relationship between the immune system and tumor formation is complicated, the action of IL10 as an immune suppressor may have different effects at different stages of cancer progression. However, the molecular mechanisms underlying the different impacts of IL10 on different cancer types remain unclear and must be further investigated.

In addition to the effect of IL10 on the tumor microenvironment through the inhibition of immune activity, IL10 also directly promotes the survival of cancer cells [27]. Here, *IL10* knockout not only decreased JAK1 activity but also inhibited Src phosphorylation, suggesting that IL10 from cancer cells or the tumor microenvironment increases JAK1 and Src activities. The interplay between the JAK/STAT pathway and Src family kinases (SFK) has been previously addressed, and full activation of STAT requires both JAK and SFK [28]. In v-Src-transformed NIH3T3 cells, STAT3 phosphorylation by Src is required for Src-induced transformation [29]. In a v-Src-transformed human gallbladder adenocarcinoma cell line, JAK2 is constitutively activated [30], suggesting that Src is involved in JAK activation. Moreover, under IL3 treatment, JAK kinase phosphorylation is mediated by v-Src and c-Src phosphorylation, and c-Src interactions with STAT3 are induced through the binding of IL3 to its receptor in normal hematopoietic cells [31]. Src has also been confirmed as an important promoter of the JAK/STAT pathway. Src is required for progestin-induced and basic fibroblastic growth factor-induced STAT3 phosphorylation in breast cancer cells and human umbilical vein endothelial cells, respectively [32, 33]. Our results consistently indicate that Src inhibition reduces JAK1 activity. However, we also observed that JAK1 inhibition and IL10 knockout reduced Src phosphorylation, indicating that Src regulates JAK1 activation and JAK1 regulates Src activation. In summary, IL10 triggers positive feedback between JAK1 and Src to amplify the activity of STAT3, thus leading to tumor formation.

Thus far, conclusive evidence has not been reported for the involvement of JAK family kinases in Src phosphorylation. Here, we found that JAK1 inhibition reduced IL10-induced Src phosphorylation, suggesting that IL10-activated JAK1 is involved in Src phosphorylation. As shown in Figure 5, Src (Y419A) failed to associate with IL10RA under IL10 treatment, indicating that IL10-mediated Src phosphorylation is essential for the recruitment of Src. Furthermore, Src inhibition also failed to promote IL10-induced signaling, which resulted in increased EGFR expression. These results indicate that both the activities of JAK1 and Src contribute to IL10-mediated EGFR expression. In particular, Src has been reported to maintain EGFR levels by inducing c-Cbl destruction, which induces EGFR ubiquitination after EGF treatment [34]. This result suggests that IL10 most likely induces EGFR expression through Src-mediated c-Cbl degradation. Moreover, Src is involved in the AP-1-induced transcription of EGFR [35], which might explain the increased EGFR mRNA levels by IL10. Finally, in addition to canonical signaling, EGF is a critical stimulator of interleukin secretion. EGF treatment and EGFR activation by PGE2-activated EP3 receptor increase IL6 production [36, 37], and EGF also increases IL8 production through KRAS activation and PI3K/Akt/Erk signaling [3, 38].

Based on our data and the result of previous studies, we suggest that IL10 activates IL10RA to recruit JAK1 and induce its autophosphorylation. The activation of JAK1 increases Src phosphorylation and subsequently interacts with IL10RA, and the positive feedback increases JAK1 phosphorylation. These results indicate for the first time that the IL10-induced activation and recruitment of Src to IL10RA is crucial for increasing JAK1/STAT3 signaling activity, which results in the expression of several downstream genes, including EGFR. Nevertheless, direct evidence to clarify whether STAT3 directly regulates EGFR expression is lacking although IL6 has been shown to increase STAT3 phosphorylation and subsequent EGFR upregulation [39]. The mechanism underlying the binding of Stat3 to the EGFR promoter to increase EGFR expression must be further elucidated. The microarray analysis revealed that the expression of other types of receptors in addition to that of EGFR are decreased in IL10 knockout mice, including ACVRL1, which encodes type I receptor of TGFβ; IGF-1 receptor; FLT1, which encodes vascular endothelial growth factor receptor 1; platelet-derived growth factor receptor alpha subunit; and IL6 receptor (Figure 5A). These results suggest that IL10 not only induces JAK1/STAT3 signaling but also cross-talks with other receptor-mediated signaling pathways to potentially promote cancer development through the activation of multiple signaling pathways. Finally, because IL10 deprivation abolishes lung cancer formation by inhibiting the formation of the microenvironment and the expression of EGFR, the future development of anti-IL10 compound(s) will be of benefit to lung cancer therapy.

## MATERIALS AND METHODS

### Collection of human specimens

The experiments using human specimens were approved by Clinical Research Ethics Committee at National Cheng Kung University Medical Center (Tainan, Taiwan). All specimens of non-small cell lung cancer were provided by the Tissue Bank, Research Center of Clinical Medicine, National Cheng Kung University Hospital (Tainan, Taiwan). Excised tissues were homogenized for Western blotting. The histological slides were used for IHC and immunofluorescent staining. The scores of pathological images were defined by the pathological scientist. The serum samples of normal people and lung cancer patients were provided by Taiwan Lung Cancer Tissue/Specimen Information Resource Center in National Health Research Institute of Taiwan (Hsinchu, Taiwan).

### Animal experiments

All animal studies were approved by the Institutional Animal Care and Use Committee at National Cheng Kung University. Scgb1a1 (secretoglobin, family 1A, member 1)-rtTA (reverse tetracycline trans-activator), TetO-Kras4b^G12D^, TetO-EGFR^L858R^ and IL10^−/−^ mice were purchased from the Jackson Lab (Bar Harbor, MA, USA). After mating, we acquired Scgb1a1-rtTA/TetO-Kras4b^G12D^, Scgb1a1-rtTA/TetO-EGFR^L858R^, Scgb1a1-rtTA/TetO-Kras4b^G12D^/IL10^−/−^ and Scgb1a1-rtTA/TetO-EGFR^L858R^/IL10^−/−^ mice. Genotyping was performed according the instruction provided by the Jackson Lab (Bar Harbor, Maine, USA). The doxycycline (Dox; 0.5 gram/liter)-induced lung tumor development was confirmed previously [40, 41]. Kras4b^G12D^ was oral administrated with Dox (Sigma-Aldich, St. Louis, MO, USA) for 5 months; EGFR^L858R^ was for 1 month to develop lung tumors. Lungs were excised for histological analysis and Western blotting. For inhibiting EGFR, mice, which orally administrated with Dox for 2 weeks, were intraperitoneally injected with gefitinib (20 mg/kg, twice/week; Sigma-Aldrich) for 4 weeks; mice were injected intraperitoneally with the anti-IL10 antibody (100 μg/mouse, once/week; eBioscience, Inc., San Diego, CA, USA) for 4 weeks to neutralize IL10.

### Histological staining

Excised lungs were fixed with 4% formaldehyde for 48h, and embedded in paraffin for slide preparation. Before staining, 5-μm slides were dewaxed by xylene and rehydrated by a graded series of ethanol. The sections were stained by hematoxylin and eosin (HE) to evaluate pathogenesis. The procedure of IHC was described in the previous study [40, 41]. Ten % BSA-blocked slides were incubated with the primary antibody targeting IL10 (Bioss Inc., Woburn, MA, USA), CD163 (abcam^®^, Cambridge, MA, USA), EGFR (Cell Signaling Technology, Beverly, MA, USA) and p-Src (Y419; GeneTex, Inc., Irvine, CA, USA) for 1h, at room temperature. Subsequent procedures were performed using Vectastain ABC kit (Vector Laboratories, Burlingame, CA) according to the instruction, and photographed by photomicroscope (Olympus, Melville, NY, USA).

### Enzyme-linked immunosorbent assay (ELISA)

IL10 concentration in human and mouse serum was measured using eBioscience ELISA kits, including 88-7106 for human and 88-7105 for mouse. The measurement was performed according to the manufacture's instruction.

### Western blotting

The blocked protein-bound PVDF was incubated with the primary antibody at 4°C overnight, including the antibody targeting IL10 (Bioss), p-Akt (Cell Signaling Technology), β-actin (Sigma-Aldrich), nucleolin (Santa Cruz Biotechnology, Inc., Dallas, Texas, USA), p-JAK1 (T1022/1023; Cell Signaling Technology), JAK1 (Cell Signaling Technology), p-STAT3 (Y705; Cell Signaling Technology), STAT3 (Santa Cruz Biotechnology Inc.), p-Src (Y419), Src (Cell Signaling Technology), Akt (Cell Signaling Technology), p-Erk (Cell Signaling Technology), Erk (Cell Signaling Technology), IL10RA, HA, GFP and EGFR (Cell Signaling Technology).

### Statistical analysis

To compare the statistical difference between 2 groups, student's *t* test was employed. The difference of 2 curves (Figure 2E and 2F(d)) were analyzed by two way analysis of variance (ANOVA). The 2 survival curves of Kaplan-Meier analysis were compared by Log-rank test. The *p*-value smaller than 0.05 was considered as the significant difference.

## SUPPLEMENTARY INFORMATION FIGURES AND TABLES


