# Supplementary material for: Positive feedback regulation between IL10 and EGFR promotes lung cancer formation
Source: Oncotarget. 2016 Mar 3;7(15):20840–54. doi: 10.18632/oncotarget.7894 (PMC4991496; doi:10.18632/oncotarget.7894)
Supplement: Supplementary file 1 [file oncotarget-07-20840-s001.pdf]

SUPPLEMENTARY INFORMATION

SUPPLEMENTARY MATERIALS AND METHODS

Reverse transcription-polymerase chain reaction (RT-PCR)

RNA was extracted using TRIsure (Bioline Inc., Taunton, MA, USA) and reversely transcripted using SuperScript II (Invitrogen). The PCR primers targeting mouse IL10 were as follows: F: 5′-tttacctggtagaagtgatgc-3′, R: 5′-cttgtagacaccttggtcttg-3′.

RNA stability and quantitative PCR (qPCR)

To evaluate RNA stability, cells were treated with 2 μM actinomycin D, and harvested as indicated times. After reverse transcription, cDNA was subjected to real time qPCR using SyBRGreen (Takara Bio, Shiga, Japan) by CFX96 REAL-TIME PCR DET SYS (Bio-Rad Laboratories, Hercules, CA, USA).

Luciferase reporter assay

To analyze the promoter activity of IL10, 1.5 kb promoter was constructed by PCR using following primers: F-5′-cctccatctgggtccatggc-3′, R-5′-ctcgagagaccttcacctctctgt-3′. After TA cloning, the promoter was ligated into pGL2 using KpnI and XhoI (New England Biolabs, Ipswich, MA, USA). For reporter assay, cells were transfected with pGL2 or pGL2-IL10 promoter for 24 h in serum-free media. Transfected cells were treated with EGF (PeproTech Asia, Rehovot, Israel) for 24 h, and harvested for reporter assay by luminometer (LB9506; Berthold Technologies, Bad Wildbad, Germany).

Colony formation assay

Five hundred cells were seeded in a well of 6-well culture plate and treated with IL10 or phosphate-buffered saline (PBS; Sigma-Aldrich) (control). Media was renew once 2 days until 10 days. To detect cell colonies, cells were stained with Methyl-blue (Sigma-Aldric) and washed once by PBS. Stained plates were air-dried overnight for subsequent photography.

Immunoprecipitation

For immunoprecipitation assay, cells were lysed in Buffer A (HEPES 10 mM, pH7.9, KCl 10 mM, $MgCl_2$ 1.5 mM, sucrose 0.34M, glycerol 10% and dithiothreitol 1mM) freshly added protease inhibitor (EMD Millipore) and phosphatase inhibitor cocktails (Biovision Inc., Milpitas, CA, USA). After centrifugation at 3000 rpm for 5 min, supernatant was subjected to immunoprecipitating using anti-Src (Cell Signaling Technology), anti-HA (Roche Diagnostics Ltd. Risch, Switzerland) and anti-GFP antibodies (OriGene Technologies, Rockville, MD, USA). After incubation at 4°C for 1 h by rotation, protein agarose beads (EMD Millipore) were added into the mixture and incubated for additional 1 h. Immune-complex was analyzed by Western blotting.

Immunofluorescence

For human and mouse tissues, after dewaxing and rehydrating, histological slides were antigen retrieval for non-stop 20 min at 100°C. Ten % BSA (Sigma-Aldrich)-blocked slides were incubated with the primary antibody targeting IL10, CD68 (Novus Biologicals, Littleton, CO, USA), CD4 (Santa Cruz Biotechnology Inc.) and FoxP3 (Invitrogen) for 1 h at room temperature. Subsequently, slides were incubated with goat anti-rabbit, anti-rat or anti-mouse IgG conjugated with Alexa Fluor® 488 or 568 (Invitrogen). Four percentage of paraformaldehyde-fixed cells were permeabilized by 0.5% triton x-100 and blocked by 10% BSA for 1 h. Blocked slides were incubated with the primary antibody targeting IL10RA or p-Src (Y419) for 1 h at room temperature. After incubation with the secondary antibody, slides were mounted by 90% glycerol containing DAPI and photographed.

Collection of EGF-treated cells-derived conditional media and treatment

Before treatment with EGF, cells were serum starved for 24 h. After EGF treatment, media was replaced with new media, and cells were incubated for additional 24 h. Finally, media was collected for subsequent experiments. To evaluate effect of conditional media on proliferation, cells were serum starved for 12 h and cultured in conditional media for the indicated time in the presence or absence of IL10 neutrolization antibody (eBioscience, San Diego, CA, USA). Control group was cultured in serum-free media.

Mutagenesis

HA-Src and GFP-IL10RA were served as templates of mutagenesis for HA-Src (Y419A) and GFP-IL10RA

(Y496A; Y446A+Y496A), respectively. Primers were listed in *Supplementary Table S3*. Mutagenesis PCR was conducted using plaque-forming unit DNA polymerase (Agilent Technologies, Santa Clara, CA, USA).

Microarray analysis

The microarray results of H1299 cells with GFP or GFP-nucleolin overexpression were performed previously [1]. Lung tissues from Scgb1a1-rtTA/TetO-EGFR^{L858R} and Scgb1a1-rtTA/TetO-EGFR^{L858R}/IL10$^{-/-}$ with doxycycline administration for 1 month were homogenized for RNA extraction using TRIsure. After reverse transcription, cDNA was subjected to microarray analysis performed by Phalanx Biotech Group (Hsinchu, Taiwan). The function group related to proliferation was extracted by Gene Set Enrichment Analysis (GSEA).

Cell culture, transfection, treatments and chemicals

Human lung cancer cell lines, A549 and H1299, were purchased from American Type Culture Collection (Manassas, VA, USA). Mouse Lung primary cells were isolated as described previously [2]. All cell lines were cultured in Dulbecco's modified Eagle's medium containing 10% fetal bovine serum, 100 µg/ml streptomycin sulfate and 100 U/ml penicillin G sodium at 37°C and 5% CO_2. PP1, the Src inhibitor and the Jak inhibitor were purchased from EMD Millipore (Billerica, MA, USA). Transfection of H1299 cells with indicated plasmids using Lipofectamine 2000 (Invitrogen Life Technologies, Grand Island, NY, USA) according to the manufacture's instruction. IL10 recombinant protein was purchase from Cell Guidance Systems LLC (Carlsbad, CA, USA).

REFERENCES

1. Hsu TI, Lin SC, Lu PS, Chang WC, Hung CY, Yeh YM, Su WC, Liao PC, Hung JJ. MMP7-mediated cleavage of nucleolin at Asp255 induces MMP9 expression to promote tumor malignancy. Oncogene. 2015; 34:826-837.

2. Hsu TI, Chen YJ, Hung CY, Wang YC, Lin SJ, Su WC, Lai MD, Kim SY, Wang Q, Qian K, Goto M, Zhao Y, Kashiwada Y, Lee KH, Chang WC, Hung JJ. A novel derivative of betulinic acid, SYK023, suppresses lung cancer growth and malignancy. Oncotarget. 2015; 6:13671-13687.

SUPPLEMENTARY FIGURES AND TABLES

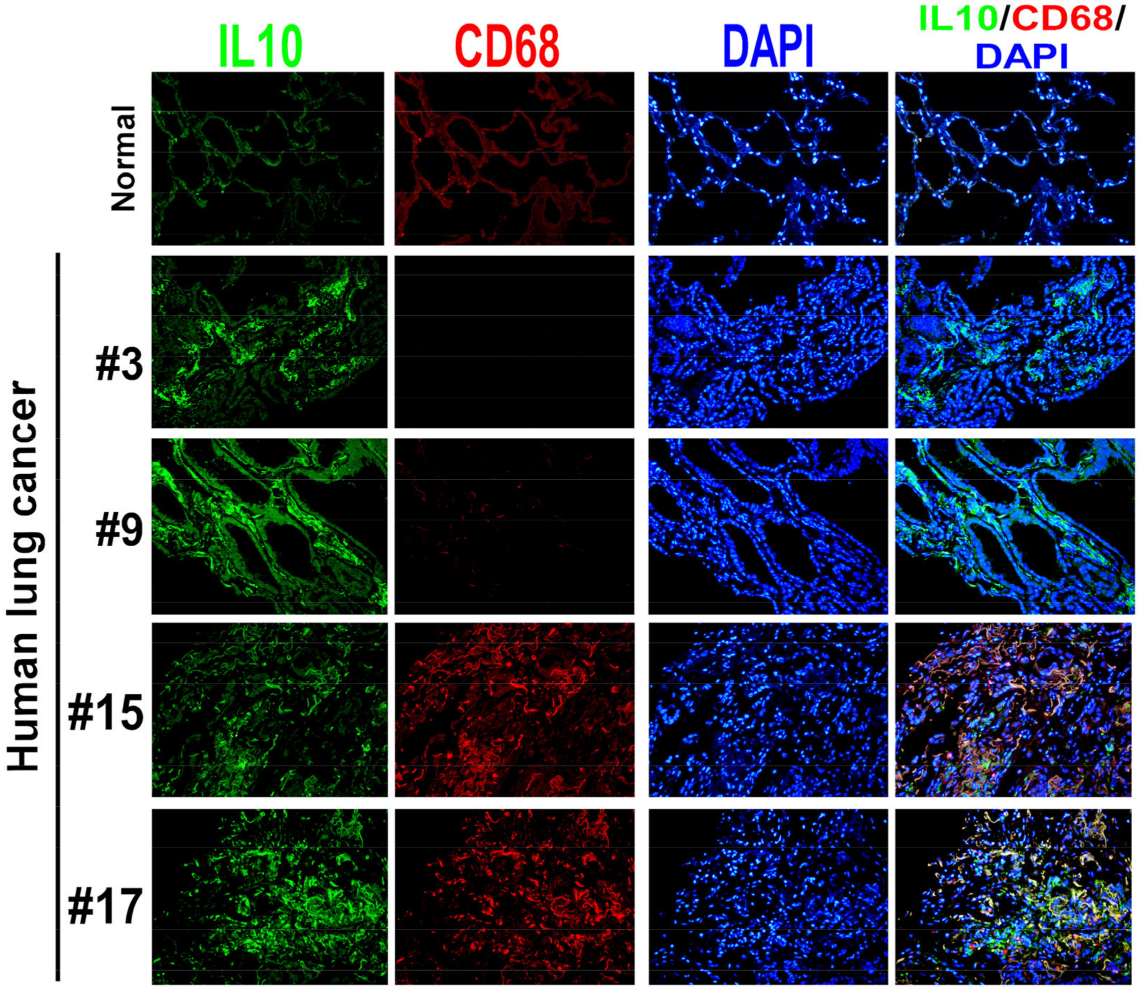

Supplementary Figure S1: IL10 expression in lung normal tissue and lung cancer. After dewaxing and rehydrating, histological slides were antigen retrieval. Blocked slides were stained with anti-IL10 and anti-CD68 antibodies. After incubation with the secondary antibody-conjugating fluorescence, slides were mounted by glycerol-containing DAPI for photographed.

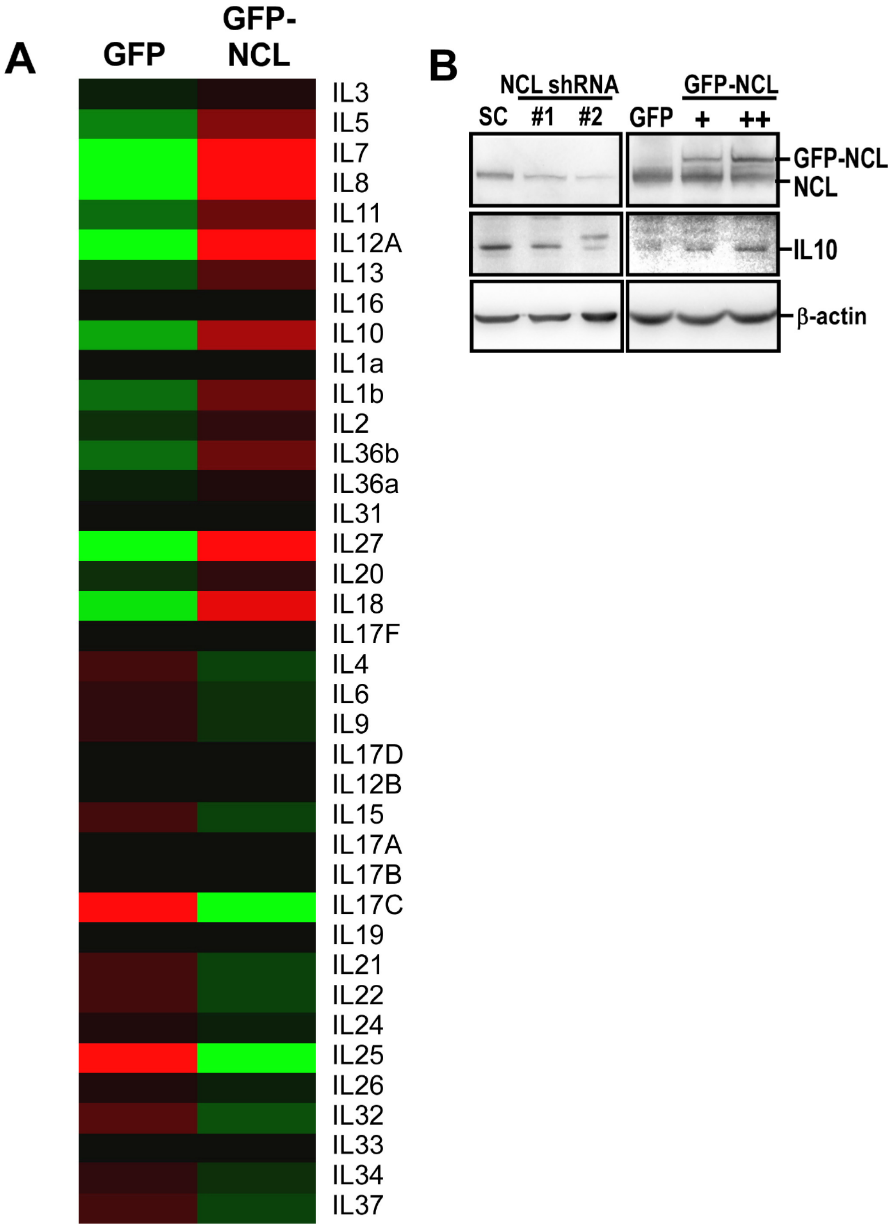

Supplementary Figure S2: The microarray analysis of H1299 cells with GFP or GFP-nucleolin (NCL) overexpression. **A.** The heatmap of microarray results. **B.** After NCL knockdown or overexpression, cells were harvested for Western blotting to detect NCL, IL10 and β-actin.

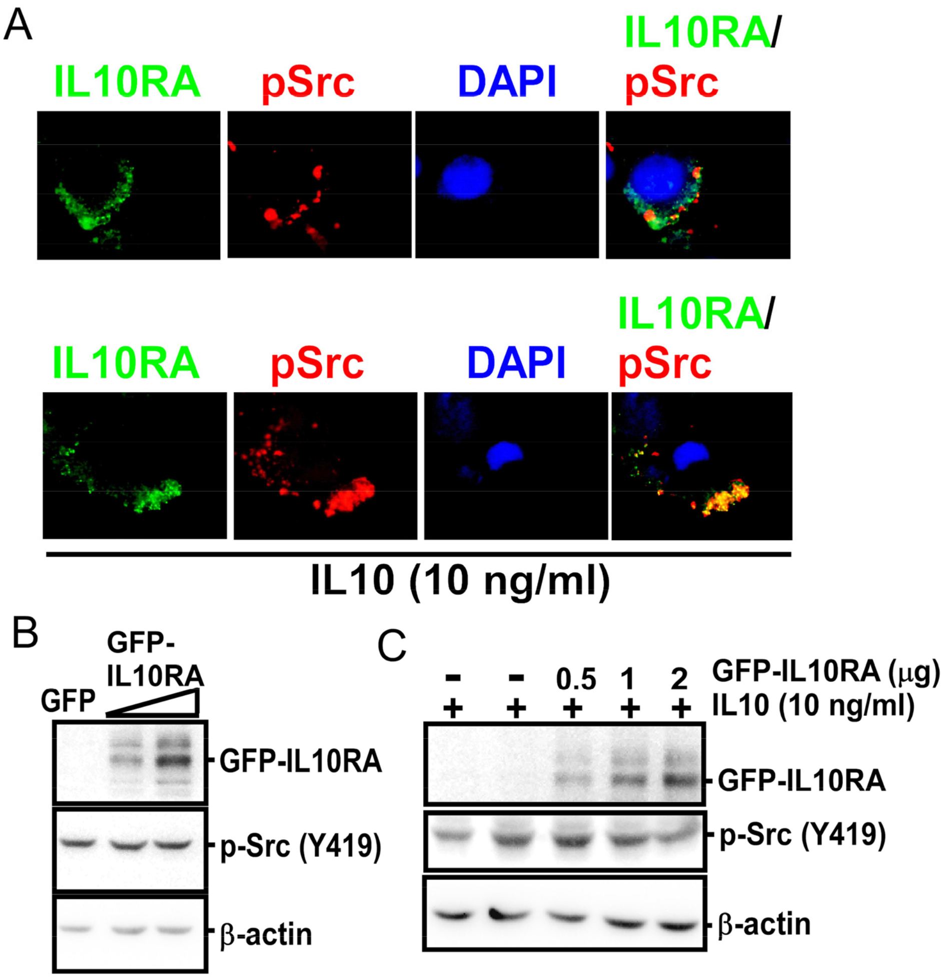

Supplementary Figure S3: The interaction of IL10RA with phospho-Src. A. After IL10 treatment for 24 h, cells were fixed and permeabilized. Blocked cells were immunofluorescently stained by antibodies targeting IL10RA and phospho-Src. **B.** After transfection with GFP-IL10RA, cells were lysed for Western blotting using the antibody targeting GFP or phospho-Src (Y419). **C.** After transfection and treatment, cells were lysed for Western blotting using the antibody targeting GFP or phospho-Src (Y419).

Supplementary Table S1: The normal people and lung cancer patients which donate the serum for measuring IL10 concentration

	Normal people	Lung cancer patients
**Number of patients**	60	60
**Gender**		
Male	38	38
Female	22	22
**Age**	60.2	62.2
**Adenocarcinoma**	None	49
**SCC**	None	11
**Stages**		
I	None	21 (IL10=33.69)
II	None	9 (IL10=34.89)
III	None	3 (IL10=46.5)
IV	None	27 (IL10=41.81)
**IL10 (pg/ml)**	32.55	38.16

Supplementary Table S2: The correlation of IL10 expression with EGFR status

EGFR status	IL10 positive	IL10 negative	Significant
**Wild type**	8	8	No
**Mutations**	11	12	
Exon 19 deletion	6	5	
L858R	5	7	

Supplementary Table S3: Primers used for site-directed mutagenesis

Genes	Primers
HA-Src (Y419A)	F: 5′-TGAAGACAATGAG**GC**CACGGCGCGGCAAGG-3′
	F: 5′-CCTTGCCGCGCCGTG**GC**CTCATTGTCTTCA-3′
GFP-IL10RA (Y496A)	F: 5′-GCAGTGACCTGGGAA**GC**TCTGACTGGAGCTTTG-3′
	F: 5′- CAAAGCTCCAGTCAGA**GC**TTCCCAGGTCACTGC-3′
GFP-IL10RA (Y446A)	F: 5′-CCTGGCCAAGGGC**GC**TTTGAAACAGGATCCTC-3′
	F: 5′-GAGGATCCTGTTTCAAA**GC**GCCCTTGGCCAGG-3′
